# "GOLD or lower limit of normal definition? a comparison with expert-based diagnosis of chronic obstructive pulmonary disease in a prospective cohort-study"

**DOI:** 10.1186/1465-9921-13-13

**Published:** 2012-02-06

**Authors:** Gülmisal Güder, Susanne Brenner, Christiane E Angermann, Georg Ertl, Matthias Held, Alfred P Sachs, Jan-Willem Lammers, Pieter Zanen, Arno W Hoes, Stefan Störk, Frans H Rutten

**Affiliations:** 1 Julius Center for Health Sciences and Primary Care, University Medical Center Utrecht, Utrecht, The Netherlands; 2 Comprehensive Heart Failure Center, University Würzburg, Department of Internal Medicine I, Würzburg, Germany; 3 Missionsärztliche Klinik, Department of Internal Medicine, Würzburg, Germany; 4 Department of Pulmonary Diseases, Heart Lung Center Utrecht, University Medical Center Utrecht, Utrecht, The Netherlands

**Keywords:** COPD diagnosis, lower limit of normal, GOLD, validation

## Abstract

**Background:**

The Global initiative for chronic Obstructive Lung Disease (GOLD) defines COPD as a fixed post-bronchodilator ratio of forced expiratory volume in 1 second and forced vital capacity (FEV1/FVC) below 0.7. Age-dependent cut-off values below the lower fifth percentile (LLN) of this ratio derived from the general population have been proposed as an alternative. We wanted to assess the diagnostic accuracy and prognostic capability of the GOLD and LLN definition when compared to an expert-based diagnosis.

**Methods:**

In a prospective cohort study, 405 patients aged ≥ 65 years with a general practitioner's diagnosis of COPD were recruited and followed up for 4.5 (median; quartiles 3.9; 5.1) years. Prevalence rates of COPD according to GOLD and three LLN definitions and diagnostic performance measurements were calculated. The reference standard was the diagnosis of COPD of an expert panel that used all available diagnostic information, including spirometry and bodyplethysmography.

**Results:**

Compared to the expert panel diagnosis, 'GOLD-COPD' misclassified 69 (28%) patients, and the three LLNs misclassified 114 (46%), 96 (39%), and 98 (40%) patients, respectively. The GOLD classification led to more false positives, the LLNs to more false negative diagnoses. The main predictors beyond the FEV1/FVC ratio for an expert diagnosis of COPD were the FEV1 % predicted, and the residual volume/total lung capacity ratio (RV/TLC). Adding FEV1 and RV/TLC to GOLD or LLN improved the diagnostic accuracy, resulting in a significant reduction of up to 50% of the number of misdiagnoses. The expert diagnosis of COPD better predicts exacerbations, hospitalizations and mortality than GOLD or LLN.

**Conclusions:**

GOLD criteria over-diagnose COPD, while LLN definitions under-diagnose COPD in elderly patients as compared to an expert panel diagnosis. Incorporating FEV1 and RV/TLC into the GOLD-COPD or LLN-based definition brings both definitions closer to expert panel diagnosis of COPD, and to daily clinical practice.

## Introduction

Chronic obstructive pulmonary disease (COPD) is among the leading causes of disability and death in developed countries. The prevalence of COPD is still on the rise, and costs for the health system are substantial [1,2]. Airflow limitation that is not fully reversible after bronchodilator application is a key feature of COPD, and spirometry is the routine diagnostic procedure of choice recommended to diagnose COPD [3,4]. However, the degree of obstruction that establishes the diagnosis of COPD is still under debate [5]. The Global Initiative for chronic Obstructive Lung Disease (GOLD) defined COPD as a fixed post-bronchodilator ratio of forced expiratory volume in 1 second and forced vital capacity (FEV1/FVC ratio) of less than 0.70 [6]. This definition is widely accepted, mainly because of its practicability.

Since the FEV1 value decreases more quickly with age than the (F)VC, the GOLD definition tends to overdiagnose COPD in the elderly [7,8]. Therefore, some authors suggested using the lower limit of normal (LLN) procedure to diagnose COPD [5]. The LLN is based on age-stratified pre-bronchodilator cut-off values of the FEV1/FVC ratio, and a value below the lower fifth percentile of an aged-matched healthy reference group is considered abnormal and consistent with a diagnosis of COPD [9,10]. Multiple studies showed that application of any population-derived LLN will result in lower prevalence estimates of COPD compared to the GOLD definition in the elderly [5,9]. The question remains however which method should be preferred. Another question is, whether there are other pulmonary function test variables that could improve the diagnostic accuracy of GOLD or LLN. The final question is, which definition predicts best prognosis and thus is useful for treatment decisions in COPD. To answer all three questions, the two criteria should be compared with an alternative, and acceptable reference standard, applied in the relevant domain, that is, a population suspected of COPD [11]. An expert panel diagnosis of COPD, based on all available diagnostic information from the clinical assessment, smoking habits, and a complete pulmonary function test (PFT) could be regarded as such a reference standard [11].

To the best of our knowledge, our study is the first to validate GOLD and LLN criteria against an expert panel diagnosis in patients suspected of COPD and to assess their prognostic ability.

Since, in daily practice, establishing the diagnosis of COPD is usually not based on a single PFT parameter, we furthermore assessed whether the addition of other PFT parameters to the GOLD or LLN criteria increases diagnostic accuracy compared to either definition alone.

## Methods

### Subject and Study Design

In a prospective cohort study, 405 patients aged ≥ 65 years with a general practitioner's (GP) diagnosis of COPD were enrolled in a stable phase of their disease. Primary assessment was between 2001 and 2003. Population and study characteristics have been published previously [12,13]. In brief, all patients underwent a detailed standardized clinical examination at an outpatient clinic (University Medical Center Utrecht) including PFT, chest radiography, and echocardiography. The study complied with the Declaration of Helsinki, and the Medical Ethical Committee of the University Medical Center Utrecht approved the study protocol. All participants gave their written informed consent.

### Pulmonary function tests

All PFT were performed with a fixed volume bodyplethysmograph and Masterscreen (Masterlab Jaeger, Würzburg, Germany). The post-bronchodilator test was assessed after inhalation of ipratropium bromide (40 micrograms twice). For predicted values of lung function markers, the recommendations of the European Respiratory Society were used [14].

### Expert panel diagnosis of COPD

In absence of a true reference standard, a consensus of an expert panel is widely accepted as the best alternative [11]. The initial expert panel of the study was composed of a qualified pulmonologist (JWL) and a GP with special interest in COPD (FHR). The panel determined presence or absence of COPD on all available results from the clinical assessment, including history taking and smoking history, chest radiographs, and finally spirometric and bodyplethysmographic information (data and graphs). Besides FEV1/FVC ratio other parameters from the PFT were also considered, including the shape of the curve, FEV1 as % predicted, presence of reversibility, RV/TLC, resistance, air trapping, and DLCO value. Also, smoking habits, a history of allergy or hyperreactivity, initiation of (periods of) dyspnoea and coughing at an early age, and a history of pulmonary embolism or lung diseases other than COPD were used in case of doubt and when applicable.

The same members of the expert panel re-evaluated the diagnosis in a random sample of 80 (20%) cases in 2011, resulting in an excellent kappa statistic between initial and repeat evaluation of 0.90. Another random sample of 120 (30%) cases was externally validated by a panel composed of a German pulmonologist (MH) and a Dutch GP with a special interest in COPD (APS). Kappa statistic between the initial panel diagnosis and that of the external panel was 0.76.

Both panels followed the aforementioned 'strategy' to diagnose COPD. Asthma was diagnosed if a reversible obstruction went along with a typical history of asthma (allergy, hyperreactivity, onset of symptoms at a young age). Reversibility was considered present if the FEV1 levels increased by 200 ml and/or 12% after bronchodilator therapy, accordingly to the American Thoracic society definition [10].

### Diagnosis of COPD according to the GOLD and LLN criteria

A post-bronchodilator FEV1/FVC ratio < 0.70 established the diagnosis 'GOLD-COPD' [4]. The 'GOLD-COPD' was graded using post-bronchodilator % of predicted FEV1 values: GOLD stage 1 (mild): ≥ 80%; stage 2 (moderate): 50-79%; stage 3 (severe): 30-49; stage 4 (very severe) < 30% [4].

A FEV1/FVC ratio below the lower fifth percentile of healthy reference groups (similar age) established the diagnosis 'LLN-COPD'. From several LLN equations provided by http://www.spirxpert.com/controversies/workinggroup.html[14-17] we selected three LLN reference equations on the basis of sample size, popularity and comparability with the age of our study population. These equations were derived from the following populations:

1. Enright et al. [16]: USA; healthy Caucasians with no respiratory symptoms, N = 1,227, 26% male, age range 65-85 years, non-smokers or all-time smoking duration < 5 years.

2. Quanjer et al. (ECCS/ERS) [14]: Europe; healthy never-smokers with no respiratory symptoms, N = 1,204, 27% male, age range 20-70 years.

3. Falaschetti et al. (Health Survey for England) [17]: Great Britain; healthy never-smokers with no respiratory disease, N = 6,053, 41% male, age range 16-85 years.

The aforementioned reference equations of the LLN-COPD definitions are based on pre-bronchodilator values, and the GOLD-COPD definition on post-bronchodilatory FEV1/FVC values. We analysed both pre- and post-bronchodilator cut-off values for both definitions. Because the results were similar, we only present the post-bronchodilator results.

### Prognostic outcomes

Exacerbations of COPD (need for short course of oral steroids), hospitalization for COPD, and all-cause mortality were assessed blinded to the diagnostic classification.

### Data analysis

**Figure 1:**
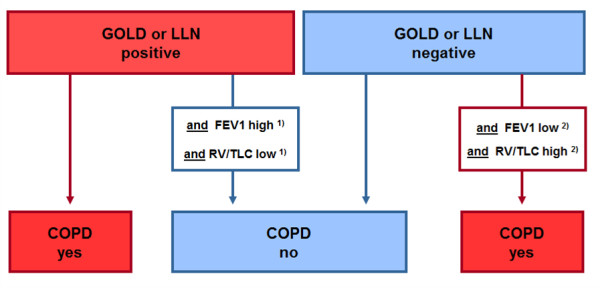
**Flow chart for diagnostic algorithm**. In clinical practice the diagnosis of COPD is based on multiple variables. As the simplest model we chose a three PFT parameters approach in which an initial COPD YES/NO diagnosis based on FEV/FVC levels was corrected if FEV1 and RV/TLC levels were altered counterintuitively*. * As thresholds for FEV1 and RV/TLC levels different cut-off levels were used and kappa statistics calculated for all alternatives. Each change in COPD diagnosis only materializes if both parameters deviate by ≤ 5/7.5/10/12.5/15/20% from 100% of the predicted value. Example: If deviations of 10% (from 100%) are chosen as thresholds for both FEV1 and RV/TLC (as % of predicted) in order to change the GOLD-COPD diagnosis from ^1) ^'yes' into 'no' (i.e., FEV1 ≥ 90% and RV/TLC ≤ 110%; [^2) ^or vice versa, from 'no' into 'yes', FEV1 < 90% and RV/TLC > 110%]), then the number of misclassified patients (false positives + false negatives) is reduced from 69 to 33, and κ- statistics improve from 0.64 to 0.83. Abbreviations: as in table 1.

Continuous data are expressed as mean (standard deviation, SD) or median (quartiles), as appropriate. Comparisons between groups were made with Fisher's exact test or Mann-Whitney *U*-test. Sensitivity, specificity, positive and negative predictive values and kappa (*κ*) statistics with 95% confidence intervals (CI) were calculated for each COPD definition with the 'expert panel diagnosis of COPD' of the initial panel as the reference test. The classification system proposed by Landis and Koch was used to determine the level of concordance (a *κ *of 0.81-1.00 is considered almost perfect) [18]. A bootstrap method was used for calculating 95% CI of *κ *and to assess statistical significance for correlated *κ *[19,20]. The diagnostic ability of different PFT parameters for predicting COPD according to the reference standard was tested using ROC curve with C-statistics with 95% CI [20]. The two PFT parameters predicting best according to the C-statistics were incorporated into a new 'modified' GOLD- or LLN-based definition, and diagnostic performance (false positives, false negatives, kappa) of these extended models were compared to the original definitions (Figure 1).

Prognostic analysis for different outcomes (exacerbation, hospitalization, death) were calculated with univariate Cox regression.

### Missing data and statistical analyses

Very few values in the dataset were missing, with the exception of the diffusion capacity of carbon monoxide (DLCO) with 42 missings. On residual volume (RV) and total lung capacity (TLC) we had five missings. As deletion of subjects with missing values may lead to biased results we imputed missing values using a regression method with the addition of a random error term [21].

All statistical analyses were carried out using the statistical software package of SPSS (PASW Statistics 18) and R for windows (version 2.11.0).

## Results

The mean age of patients was 73 (5.3) years, 45% were female. The baseline characteristics of participants are shown in table 1 and 2. Table 3 shows the differential performance of the GOLD approach and of three different LLN definitions with the expert panel diagnosis as the reference test.

**Table 1:** Baseline characteristics of patients with and without COPD according to the expert panel and the GOLD definition

	All Subjects	Expert Panel		P	GOLD definition		P
					
	(n = 405)	No COPD (n = 158)	COPD (n = 247)		No COPD (n = 161)	COPD (n = 244)	
Age, years	72(69; 77)	71.5(67; 76)	73(70; 77)	**0.004**	72(67; 76)	73(70; 77)	**0.010**
Male sex, %	55.1	40.5	64.4	**< 0.001**	34.8	68.4	**< 0.001**
Death, %	14.9	6.3	20.5	**< 0.001**	10.6	17.8	**0.047**
Pack years smoking	16.8 (0; 38.8)	1.5 (0; 23)	27 (8.3; 51)	**< 0.001**	2.3 (0; 28)	25.2 (5.5; 51)	**< 0.001**
BMI, kg/m^2^	26.2 (24.1; 28.8)	27 (24.8; 30.1)	25.6 (23.4; 28.3)	**< 0.001**	27 (24.7; 30)	25.6 (23.3; 28.3)	**< 0.001**
**Comorbidities and Symptoms**							
Hypertension, %	39.0	42.4	36.8	**0.30**	46.6	34.0	**0.013**
Diabetes, %	8.6	8.9	8.5	**1.00**	9.9	7.8	**0.47**
Stroke or TIA, %	22.0	17.1	25.1	**0.07**	18.0	24.6	**0.14**
Fatigue, %	41.3	46.8	37.8	**0.08**	46.6	37.9	**0.09**
Wheezing, %	63.5	55.1	68.8	**0.006**	59.0	66.4	**0.14**
**COPD Inhalatory Medication**							
Beta2-mimetics, %	58.2	39.9	69.9	**< 0.001**	41.6	69.1	**< 0.001**
Anticholinergics, %	47.6	36.1	55.1	**< 0.001**	36.0	55.4	**< 0.001**
Inhaled Corticosteroids, %	63.0	56.3	67.2	**0.035**	58.4	66.0	**0.14**

Data are shown as median (25^th^; 75^th ^percentile) or %, as appropriate. P-value refers to χ^2^-test or Mann-Whitney-U test as appropriate.

Abbreviations: **BMI**, body mass index, **TIA**, transient ischemic attack.

**Table 2:** Pulmonary function test of patients with and without COPD according to the expert panel and the GOLD definition

	All Subjects	Expert Panel		P	GOLD definition		P
					
	(n = 405)	No COPD (n = 158)	COPD (n = 247)		No COPD (n = 161)	COPD (n = 244)	
**Pre-bronchodilator Pulmonary Function Test**							
FEV1, %	76 (57; 96)	100 (92; 111)	62 (48; 75)	**< 0.001**	99 (84; 110)	63 (48; 79)	**< 0.001**
FVC, %	95 (78; 109)	109 (99; 120)	84 (74; 96)	**< 0.001**	104 (91; 117)	88 (76; 102)	**< 0.001**
FEV1/FVC	0.66 (0.55; 0.74)	0.75 (0.7; 0.8)	0.58 (0.48; 0.66)	**< 0.001**	0.75 (0.71; 0.8)	0.58 (0.48; 0.65)	**< 0.001**
TLC, %	110 (100; 122)	107 (98; 116)	113 (102; 125)	**< 0.001**	105 (97; 114)	114 (104; 125)	**< 0.001**
RV, %	126 (106; 159)	108 (94; 121)	146 (120; 175)	**< 0.001**	108 (96; 126)	142 (117; 174)	**< 0.001**
RV/TLC, %	114 (99; 132)	99 (92; 108)	124 (112; 140)	**< 0.001**	102 (93; 115)	122 (108; 138)	**< 0.001**
DLCO, %	72 (58; 83)	81 (71; 91)	63 (49; 76)	**< 0.001**	78 (67; 89)	66 (50; 79)	**< 0.001**
**Post-bronchodilator Pulmonary Function Test**							
FEV1, %	82 (64; 102)	106 (97; 116)	69 (56; 81)	**< 0.001**	103 (89; 115)	70 (56; 85)	**< 0.001**
FVC, %	102 (88; 117)	114 (103; 125)	92 (80; 108)	**< 0.001**	105 (91; 120)	98 (85; 115)	**0.004**
FEV1/FVC	0.66 (0.55; 0.75)	0.76 (0.71; 0.81)	0.58 (0.48; 0.66)	**< 0.001**	0.77(0.74; 0.81)	0.57 (0.47;0. 65)	**< 0.001**

Data are shown as median (25^th^; 75^th ^percentile). All PFT variables except FEV1/FVC are presented as percentage of predicted. P-value refers to Mann-Whitney-U test.

Abbreviations: **DLCO**, diffusion capacity of carbon monoxide, **FEV1**, forced expiratory volume in 1 second, **FVC**, forced vital capacity, **RV**, residual volume, **TLC**, total lung capacity.

**Table 3:** Diagnostic test performance of GOLD and LLN with the expert panel as the reference test

	GOLD	**LLN: Enright **[16]	**LLN: Quanjer **[14]	**LLN: Falaschetti **[17]
**COPD prevalence (N)**	244 (60%)	142 (35%)	167 (41%)	175(43%)
**False positives (N)**	33	6	9	9
**False negatives (N)**	36	111	89	81
**Sensitivity**	85.4%	55.1%	64.0%	67.2%
**Specificity**	79.1%	96.2%	94.3%	94.3%
**Positive predictive value**	86.5%	95.8%	94.6%	94.9%
**Negative predictive value**	77.6%	57.8%	62.6%	64.8%
**Kappa coefficient**	0.64 (0.56; 0.71)	0.46 (0.38; 0.53)	0.53 (0.46; 0.60)	0.57 (0.50; 0.64)
P		< 0.001	0.006	0.053

COPD prevalence as % or N within the total cohort (N = 405) according to different definitions. P for comparison of κ coefficients of GOLD vs LLN.

In our elderly cohort all regression equations used for the LLN definition had a lower FEV1/FVC threshold than the GOLD definition. Specificity was higher and sensitivity lower for LLN than GOLD. When compared to the reference test, kappa statistics were higher for GOLD than for any of the three LLN definitions, however not all differences were statistically significant (table 2).

### 'Misdiagnosed' patients with the GOLD definition as compared to the expert panel

There was reasonable concordance between the diagnosis of COPD with the GOLD definition and the expert panel (*κ *= 0.64, 95% CI 0.57-0.71, table 3). Classification according to GOLD resulted in 33 false positive and 36 false negative diagnoses as compared to the expert panel diagnosis of COPD (table 4).

**Table 4:** Baseline characteristics of patients with a 'correct' and 'false' GOLD-COPD diagnosis according to the reference-test

GOLD- COPD	FEV1/FVC	FEV1 [% pred]	FVC [% pred]	TLC [% pred]	RV [% pred]	RV/TLC [% pred]	DLCO [% pred]	Age [Years]	Pack years [Years]
**True** **positive**	0.56 (0.46; 0.64)	67 (54; 79)	94 (82; 110)	114 (103; 126)	147 (122; 177)	124 (112; 140)	62 (49; 76)	73 (70; 77)	28.0 (10.6; 52.5)
**N = 211**	**211**	**211**	**211**	**207**	**207**	**207**	**181**	**211**	**211**
**False** **positive**	0.66 (0.61; 0.68)	98 (94; 107)	119 (108; 133)	113 (104; 120)	119 (107; 135)	103 (94; 109)	78 (70; 91)	72 (69; 78)	3.4 (0; 22.5)
**N = 33**	**33**	**33**	**33**	**33**	**33**	**33**	**33**	**33**	**33**
**True** **negative**	0.78 (0.74; 0.83)	108 (100; 118)	112 (100; 123)	105 (97; 113)	106 (94; 116)	98 (92; 107)	82 (72; 92)	71 (67; 75)	1.1 (0; 23.8)
**N = 125**	**125**	**125**	**125**	**124**	**124**	**124**	**119**	**125**	**125**
**False** **negative**	0.74 (0.72; 0.76)	79 (71; 85)	86 (75; 93)	104 (94; 118)	135 (106; 165)	124 (113; 146)	67 (54; 74)	73 (69; 78)	19.1 (0; 40.9)
**N = 36**	**36**	**36**	**36**	**36**	**36**	**36**	**30**	**36**	**36**

COPD was defined by the GOLD definition and test results were categorized into 'correct' or 'false' according to the diagnosis of the expert-panel.

FEV1/FVC, FEV1, FVC are given as post-bronchodilator values; TLC, RV and DLCO as pre-bronchodilator values.

Abbreviations: as in table 1.

In general, patients with a "true positive COPD diagnosis" tend to have RV/TLC values (far) above 100% of predicted, a FEV1 value (far) below 100% of predicted, and a DLCO values (far) below 80% of predicted, as compared to healthy individuals.

In our cohort, the median RV/TLC of patients with COPD according to the expert panel was high (median [quartiles]: 124 [112; 140] as % of predicted), median FEV1 low (67 [54;79] % of predicted), and also DLCO levels low (62 [49;76] % of predicted) (table 4).

### Prognostic capacity of the COPD definitions

During a median follow-up of 4.5 (quartiles 3.9; 5.1) years, 148 patients experienced at least one episode of a COPD exacerbation (defined as a 7-10 days boots of prednisolone use), 67 patients were hospitalized for pulmonary reasons, and 60 patients died.

A COPD diagnosis according to the expert panel identified the largest number of patients that experienced any of the aforementioned events, followed by COPD according to GOLD and COPD-LLN (see table 5). The occurrence of outcomes related to the different classifications of COPD with percentages related to the classification of COPD is presented in table 6.

**Table 5:** Prognostic outcomes according to different COPD definitions within the whole cohort

COPD definition (N*)	Exacerbations of COPD (N = 148)	Pulmonary Hospitalizations (N = 67)	All-cause Mortality (N = 60)
Expert COPD (N* = 247)	114/148 (77.0%)	49/67 (73.1%)	50/60 (83.3%)
GOLD COPD (N* = 244)	114/148 (77.0%)	46/67 (68.7%)	43/60 (71.7%)
LLN: Enright [16] (N* = 142)	76/148 (51.4%)	34/67 (50.7%)	32/60 (53.3%)
LLN: Quanjer [14] (N* = 167)	88/148 (59.5%)	37/67 (55.2%)	35/60 (58.3%)
LLN: Falaschetti [17] (N* = 175)	91/148 (61.5%)	37/67 (55.2%)	35/60 (58.3%)

Absolute numbers (%) of clinically relevant outcomes according to the respective definition of COPD. N* corresponds to the number of patients classified as COPD according to the individual COPD definitions. The denominator corresponds to the absolute number of events.

**Table 6:** Prognostic outcomes according to different COPD definitions within each "COPD definition"

COPD definition N*	Exacerbations of COPD (N = 148)	Pulmonary Hospitalizations (n = 67)	All-cause Mortality (n = 60)
Expert COPD (N* = 247)	114/247 (46.2%)	49/247 (19.8%)	50/247 (20.2%)
GOLD COPD (N* = 244)	114/244 (46.7%)	46/244 (18.9%)	43/244 (17.6%)
LLN: Enright [16] (N* = 142)	76/142 (53.5%)	34/142 (23.9%)	32/142 (22.5%)
LLN: Quanjer [14] (N* = 167)	88/167 (52.7%)	37/167 (22.2%)	35/167 (21.0%)
LLN: Falaschetti [17] (N* = 175)	91/175 (52.0%)	37/175 (21.1%)	35/175 (20.0%)

Absolute numbers (%) of clinically relevant outcomes in patients classified as COPD according to the different definitions. N* corresponds to the number of patients classified as COPD according to the individual COPD definitions. The denominator corresponds also to the absolute number of individuals within each COPD definition.

Hazard ratios of COPD yes versus no for the prognostic outcomes for any of the definitions are presented in table 7. With all COPD definitions, those with COPD had significantly worse prognostic outcomes as compared to those with 'no COPD'.

**Table 7:** Prognostic outcomes according to different COPD definitions in univariate Cox regression analysis

COPD definition	Exacerbations of COPD HR (95% CI) P	Hospitalisation for pulmonary reason HR (95% CI) P	All-cause death HR (95% CI) P
Expert panel (N = 247)	2.94 (1.99;4.31) P < 0.001	2.03 (1.18;3.49) P = 0.010	3.59 (1.82;7.07) P < 0.001
GOLD-COPD (N = 244)	2.92 (1.99;4.28) P < 0.001	1.62 (0.97;2.72) P = 0.067	1.81 (1.03;3.17) P = 0.038
LLN: Enright [16] (N = 142)	2.70 (1.95;3.73) P < 0.001	2.20 (1.36;3.55) P = 0.001	2.28 (1.37;3.78) P = 0.001
LLN: Quanjer [14] (N = 167)	2.97 (2.14;4.14) P < 0.001	2.06 (1.27;3.33) P = 0.003	2.21 (1.32;3.70) P = 0.002
LLN: Falaschetti [17] (N = 175)	2.89 (2.07;4.03) P < 0.001	1.87 (1.15;3.03) P = 0.011	2.02 (1.21;3.38) P = 0.007

Univariate Cox Regression analysis comparing COPD yes versus no diagnosis per definition. N corresponds to the number of patients with COPD according to the different definitions of COPD.

Abbreviations:

**CI**, Confidence interval, **HR**, Hazard ratio, **FN**, false negative, **FP**, false positive, **TN**, true negative, **TP**, true positive.

### Pulmonary function test predictors of the diagnosis of COPD, using the expert panel as the reference

From all PFT variables (besides FEV/FVC), FEV1 % predicted, RV/TLC and DLCO % predicted performed best in predicting the expert diagnosis of COPD. The c-statistics of these variables using the expert panel diagnosis of COPD as the reference were 0.95 (95% CI 0.93-0.97), 0.85 (95% CI 0.81-0.89), and 0.77 (95% CI 0.73-0.82), respectively.

### Addition of FEV1 and RV/TLC to GOLD-COPD and LLN-COPD

Addition of FEV1 and RV/TLC (both as % predicted) to the GOLD- or LLN-based definition improved diagnostic test performance significantly. Kappa statistics for the GOLD definition increased from 0.64 up to 0.83 (p < 0.001) and the number of misdiagnoses decreased from 69 to 33 (highest kappa statistics seen for 10% deviation; see Figure 1 for explanation of the algorithm). For the LLN definitions (Enright/Quanjer/Falaschetti) the Kappa raised from 0.46/0.53/0.57 up to 0.77/0.79/0.80 and the number of misdiagnosis decreased from 117/98/90 to 44/40/39 (highest kappa statistics seen for all 3 LLN definitions for 5% deviation; see Figure 1 for explanation of the algorithm).

## Discussion

In our study we show that false positive diagnosis of COPD occurred more often with the GOLD definition, while false negatives were more common with the LLN definitions as compared to an expert panel diagnosis as the reference test. Adding FEV1 and RV/TLC improved the GOLD and LLN approach, reducing misdiagnosed COPD by up to 50% depending on the cut-points applied. The expert panel diagnosis predicted best the occurrence of exacerbations of COPD, pulmonary hospitalizations, and all-cause mortality, followed by the GOLD and LLN definitions.

The choice of a fixed cut-off point for the GOLD-COPD definition was made for reasons of generalization and simplification [22]. Although even lower FEV1/FVC ratios than 0.7 can be expected in the elderly without a pathological correlate, [7] a spirometric test result of > 0.7 does not necessarily exclude a diagnosis of COPD in these patients. Especially elderly patients tend to incompletely empty their lungs during the performance of the FVC manoeuvre [23], resulting in a lower FVC value and thus an increased FEV1/FVC ratio, rendering false-negative COPD diagnoses more likely.

**Figure 2:**
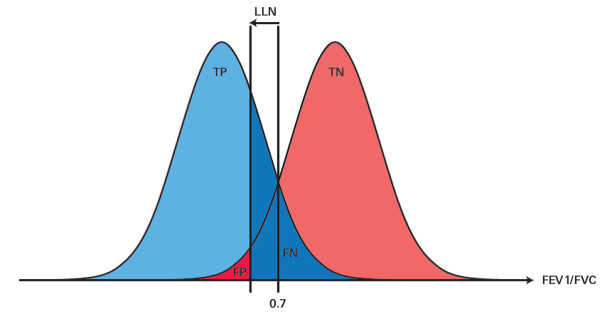
**Change of the threshold of FEV1/FVC ratio will change the amount of misdiagnosis in both directions**. Application of the LLN definition in elderly patients which generally results in FEV1/FVC levels smaller than 0.7 reduces the number of FP diagnoses but subsequently increases the FN.

Multiple studies already showed that fewer patients are diagnosed as COPD positive when LLN definitions are applied instead of GOLD, especially in the elderly (e.g., 36 vs. 15% in a healthy Dutch cohort of patients aged ≥ 50 years) [5,7,9,24,25]. The present study confirms the aforementioned differences in prevalence rates of COPD according to GOLD or LLN. Importantly, however, all previous studies involved in the discussion whether LLN or GOLD should be applied, compared both methods without application of a reference test. Without a reference, however, it is impossible to answer which method performs better [11]. This lack of evidence and the resulting diagnostic uncertainties have not been adequately appreciated. Application of the LLN will increase the chance of classifying COPD patients as having no COPD and thus the risk of undertreatment of especially elderly patients (Figure 2).

The diagnosis of our expert panel was validated internally and externally. Re-evaluation of 80 cases (20%) in 2011 by the same panel as in 2001/2003 had a very good kappa of 0.90. External validation with a panel including a German pulmonologist and a Dutch GP with special interest in COPD was somewhat lower with a kappa of 0.76, which still can be considered as a good accordance.

Despite a higher number of patients with mild COPD, the expert panel diagnosis of COPD was highly associated with COPD exacerbations, pulmonary hospitalizations and all-cause deaths, underlining the validity of the expert panel diagnosis.

As expected, a LLN-based diagnosis of COPD generated less false positives and more false negatives as compared to the conventional GOLD definition. The overall accuracy of LLN was similar or worse than the conventional GOLD definition when compared to the expert panel diagnosis of COPD. Misclassifications occurred mainly in patients with GOLD stage I and II.

Typically, in diagnostic test research there is a trade-off between specificity and sensitivity (see Figure 2). For a balanced approach the consequences of false positive and false negative cases should be assessed. A false positive result in the case of COPD may lead to over-treatment and therefore avoidable expenses for the health system. Furthermore, the adverse effects of pulmonary medication might cause more harm than benefit to some patients [26-28]. In addition, a false positive diagnosis of COPD increases the risk that physician and patient remain unaware of other possible reasons for the complaints, such as cardiovascular diseases, notably heart failure [29].

The effects of a false negative diagnosis is undertreatment of patients with COPD at a point in time when they probably would benefit most (GOLD stages I and II). Table 5 summarizes the effect of classifying the presence or absence of 'COPD' according to the different methods. LLN tends to categorize elderly with mild obstruction as 'no COPD' (high specificity and low sensitivity). In absolute numbers, LLN identified fewer patients with clinically relevant prognostic events (COPD exacerbation, hospitalization, mortality) than the GOLD or panel definition. As a clinical consequence, fewer elderly patients would receive therapy targeted at reducing these events, when the clinician would apply LLN instead of the GOLD or panel diagnoses. Table 7 shows that the prognostic abilities of LLN, GOLD and panel were compatible with clearly overlapping 95% confidence intervals of the hazard ratios. Early diagnosis and identification of false negatives may enable intervention strategies as counseling for smoking cessation and exercise training when pulmonary compromise is still mild [30]. Initiation of pharmacotherapy can reduce symptoms, improve quality of life, and decrease the number of acute exacerbations [31,32]. Guidelines therefore advocate early detection of airflow limitation [4].

A multiple test result approach with incorporating bodyplethysmographic data seems a reasonable way to establish a more reliable diagnosis of COPD, although, we have to consider that bodyplethysmography is costly, with an average prices of 75 to 200 US Dollars per performance [33].

FEV1 is probably the most important determinant of obstruction, and RV/TLC is known to be highly and inversely correlated to FEV1% of predicted [33]. Normal values of FEV1 and RV/TLC in subjects with a FEV1/FVC ratio < 0.70 should motivate re-evaluation of a positive diagnosis of COPD based solely on the conventional GOLD criteria. An approach many pulmonologists apply in clinical practice. As an alternative, DLCO could be used instead of RV/TLC, although more missing and indecisive results with this method were seen in our analysis and might be generally be expected (data not shown).

The National Institute for Clinical Excellence (NICE) acknowledged the importance of FEV1 as distinct parameter to diagnose COPD, and defined airflow obstruction if both the FEV1/FVC ratio is < 0.7 *and *the FEV1 < 80% of predicted, and thus 'starting' the diagnosis of COPD from GOLD II onwards [34]. Application of the NICE definition (post-bronchodilator values) in our cohort as compared to the expert panel diagnosis of COPD led to very high specificity (99%; only two false positives) but low sensitivity (66%, 82 false negatives). Incorporating a second PFT parameter as FEV1 into a FEV1/FVC-based definition might effectively reduce false positive test results, however for correction of false negative results at least a three parameters approach is needed.

Certain limitations need to be considered in the interpretation of our findings. PFT was only performed once at baseline, and secular trends could have been missed. Second, information on graphical PFT results as the flow volume curve or the flow pressure curve also enhance the diagnostic ability of the expert panel, but we could not quantify how much these graphs added to the final decision of the panel.

Another limitation in our study is incorporation bias when assessing the added value of other PFT variables to improve the diagnosis of GOLD or LLN [35]. PFT parameters play an important role in the diagnosis of the expert panel. Thus, overoptimism of the diagnostic performance of PFT variables such as FEV1 and RV/TLC should be considered. Robust external validation and accurate cut-off calculations are still needed before the proposed algorithm of including FEV1 and RV/TLC in the GOLD or LLN definition may be adopted in routine practice. Our intention, however, was not to create a new definition of COPD, but to raise the awareness of some of the shortcomings of the single fixed cut-off value of FEV/FVC 0.7 and the age-adjusted LLN definitions.

In **conclusion**, both the conventional GOLD criteria and some of the most frequently used LLN-based diagnoses of COPD share major shortcomings as compared to the expert panel diagnosis of COPD. While GOLD definition tends to overdiagnose COPD, LLN-based definitions tend to underdiagnose COPD in symptomatic patients. Adding the information on FEV1 and RV/TLC to the GOLD definition reduced the number of misdiagnoses substantially for either definition. Further studies are needed to explore the usefulness of 'an upgraded' COPD or LLN diagnosis with determination of the optimal cut-off values for RV/TLC and DLCO.

## Abbreviations

BMI: Body mass index; COPD: Chronic obstructive pulmonary disease; DLCO: Diffusion capacity of carbon monoxide; ECCS European Community for Coal and Steel; ERS European Respiratory Society; FEV1: Forced expiratory volume in 1 second; FVC: Forced vital capacity; GOLD Global Initiative for chronic Obstructive Lung Disease; GP: General practitioner; LLN Lower limit of normal; NICE National Institute for Clinical Excellence; PFT: Pulmonary function test; RV: Residual volume; SD Standard deviation; TIA: Transient ischemic attack; TLC: Total lung capacity; USA United States of America.

## Competing interests

The authors declare that they have no competing interests.

## Authors' contributions

GG analyzed and interpreted the data and wrote the manuscript. SB, MH, CEA and GE contributed significant intellectual content to the manuscript. APS recruited patients and contributed significant intellectual content to the manuscript. MH and APS were furthermore part of the external panel.

AWH helped to set up and design the study, and was instrumental in grant application, and contributed significant intellectual content to the manuscript. PZ supervised all pulmonary function tests and contributed significant intellectual content to the manuscript. JWL was part of the expert panel and contributed intellectual content to drafting the article. SS supervised data analysis and wrote the paper. FRH designed the study, recruited patients, interpreted the data and wrote the manuscript. All authors read and approved the final manuscript.

## References

[B1] Murray CJ Lopez AD Alternative projections of mortality and disability by cause 1990-2020: Global Burden of Disease Study Lancet 1997 349 9064 1498 1504 10.1016/S0140-6736(96)07492-2 9167458

[B2] Blanchette CM Gutierrez B Ory C Chang E Akazawa M Economic burden in direct costs of concomitant chronic obstructive pulmonary disease and asthma in a Medicare Advantage population J Manag Care Pharm 2008 14 2 176 185 18331119 10.18553/jmcp.2008.14.2.176 PMC10437934

[B3] Celli BR MacNee W Standards for the diagnosis and treatment of patients with COPD: a summary of the ATS/ERS position paper Eur Respir J 2004 23 6 932 946 10.1183/09031936.04.00014304 15219010

[B4] Rabe KF Hurd S Anzueto A Barnes PJ Buist SA Calverley P Fukuchi Y Jenkins C Rodriguez-Roisin R van Weel C Zielinski J Global strategy for the diagnosis, management, and prevention of chronic obstructive pulmonary disease: GOLD executive summary Am J Respir Crit Care Med 2007 176 6 532 555 10.1164/rccm.200703-456SO 17507545

[B5] Swanney MP Ruppel G Enright PL Pedersen OF Crapo RO Miller MR Jensen RL Falaschetti E Schouten JP Hankinson JL Stocks J Quanjer PH Using the lower limit of normal for the FEV1/FVC ratio reduces the misclassification of airway obstruction Thorax 2008 63 12 1046 1051 10.1136/thx.2008.098483 18786983

[B6] Pauwels RA Buist AS Calverley PM Jenkins CR Hurd SS Global strategy for the diagnosis, management, and prevention of chronic obstructive pulmonary disease. NHLBI/WHO Global Initiative for Chronic Obstructive Lung Disease (GOLD) Workshop summary Am J Respir Crit Care Med 2001 163 5 1256 1276 11316667 10.1164/ajrccm.163.5.2101039

[B7] Hardie JA Buist AS Vollmer WM Ellingsen I Bakke PS Morkve O Risk of over-diagnosis of COPD in asymptomatic elderly never-smokers Eur Respir J 2002 20 5 1117 1122 10.1183/09031936.02.00023202 12449163

[B8] Celli BR Halbert RJ Isonaka S Schau B Population impact of different definitions of airway obstruction Eur Respir J 2003 22 2 268 273 10.1183/09031936.03.00075102 12952259

[B9] Hansen JE Sun XG Wasserman K Spirometric criteria for airway obstruction: Use percentage of FEV1/FVC ratio below the fifth percentile, not < 70% Chest 2007 131 2 349 355 10.1378/chest.06-1349 17296632

[B10] Pellegrino R Viegi G Brusasco V Crapo RO Burgos F Casaburi R Coates A van der Grinten CP Gustafsson P Hankinson J Jensen R Johnson DC MacIntyre N McKay R Miller MR Navajas D Pedersen OF Wanger J Interpretative strategies for lung function tests Eur Respir J 2005 26 5 948 968 10.1183/09031936.05.00035205 16264058

[B11] Moons KG Grobbee DE When should we remain blind and when should our eyes remain open in diagnostic studies? J Clin Epidemiol 2002 55 7 633 636 10.1016/S0895-4356(02)00408-0 12160909

[B12] Rutten FH Cramer MJ Grobbee DE Sachs AP Kirkels JH Lammers JW Hoes AW Unrecognized heart failure in elderly patients with stable chronic obstructive pulmonary disease Eur Heart J 2005 26 18 1887 1894 10.1093/eurheartj/ehi291 15860516

[B13] Boudestein LC Rutten FH Cramer MJ Lammers JW Hoes AW The impact of concurrent heart failure on prognosis in patients with chronic obstructive pulmonary disease Eur J Heart Fail 2009 11 12 1182 1188 10.1093/eurjhf/hfp148 19887495

[B14] Quanjer PH Tammeling GJ Cotes JE Pedersen OF Peslin R Yernault JC Lung volumes and forced ventilatory flows. Report Working Party Standardization of Lung Function Tests, European Community for Steel and Coal. Official Statement of the European Respiratory Society Eur Respir J Suppl 1993 16 5 40 8499054

[B15] The Pulmonaria Group CRO Enright PL Falaschetti E Hankinson JL Jenkins C Jensen RL Miller MR Pedersen OF Quanjer PH Ruppel GL Schouten JP Stocks J Swanney MP Interpreting Spirometric Test Results http://www.spirxpert.com

[B16] Enright PL Adams AB Boyle PJ Sherrill DL Spirometry and maximal respiratory pressure references from healthy Minnesota 65- to 85-year-old women and men Chest 1995 108 3 663 669 10.1378/chest.108.3.663 7656613

[B17] Falaschetti E Laiho J Primatesta P Purdon S Prediction equations for normal and low lung function from the Health Survey for England Eur Respir J 2004 23 3 456 463 10.1183/09031936.04.00055204 15065839

[B18] Landis JR Koch GG The measurement of observer agreement for categorical data Biometrics 1977 33 1 159 174 10.2307/2529310 843571

[B19] Vanbelle SAA A bootstrap method for comparing correlated kappa coefficients Journal of Statistical Computation & Simulation 2008 78 1009 1015 10.1080/0094965070141024922319141

[B20] Carpenter J Bithell J Bootstrap confidence intervals: when, which, what? A practical guide for medical statisticians" Statistics in Medicine 2000 19 1141 1164 10.1002/(SICI)1097-0258(20000515)19:9<1141::AID-SIM479>3.0.CO;2-F 10797513

[B21] Greenland S Finkle WD A critical look at methods for handling missing covariates in epidemiologic regression analyses Am J Epidemiol 1995 142 12 1255 1264 7503045 10.1093/oxfordjournals.aje.a117592

[B22] Calverley PM The GOLD classification has advanced understanding of COPD Am J Respir Crit Care Med 2004 170 3 211 212 discussion 214 10.1164/rccm.2405008 15280171

[B23] Bellia V Sorino C Catalano F Augugliaro G Scichilone N Pistelli R Pedone C Antonelli-Incalzi R Validation of FEV6 in the elderly: correlates of performance and repeatability Thorax 2008 63 1 60 66 10.1136/thx.2007.080572 17702791

[B24] Roberts SD Farber MO Knox KS Phillips GS Bhatt NY Mastronarde JG Wood KL FEV1/FVC ratio of 70% misclassifies patients with obstruction at the extremes of age Chest 2006 130 1 200 206 10.1378/chest.130.1.200 16840402

[B25] Schermer TR Smeele IJ Thoonen BP Lucas AE Grootens JG van Boxem TJ Heijdra YF van Weel C Current clinical guideline definitions of airflow obstruction and COPD overdiagnosis in primary care Eur Respir J 2008 32 4 945 952 10.1183/09031936.00170307 18550607

[B26] Salpeter SR Ormiston TM Salpeter EE Cardiovascular effects of beta-agonists in patients with asthma and COPD: a meta-analysis Chest 2004 125 6 2309 2321 10.1378/chest.125.6.2309 15189956

[B27] Drummond MB Dasenbrook EC Pitz MW Murphy DJ Fan E Inhaled corticosteroids in patients with stable chronic obstructive pulmonary disease: a systematic review and meta-analysis JAMA 2008 300 20 2407 2416 10.1001/jama.2008.717 19033591 PMC4804462

[B28] Singh S Loke YK Furberg CD Inhaled anticholinergics and risk of major adverse cardiovascular events in patients with chronic obstructive pulmonary disease: a systematic review and meta-analysis JAMA 2008 300 12 1439 1450 10.1001/jama.300.12.1439 18812535

[B29] Rutten FH Cramer MJ Lammers JW Grobbee DE Hoes AW Heart failure and chronic obstructive pulmonary disease: An ignored combination? Eur J Heart Fail 2006 8 7 706 711 10.1016/j.ejheart.2006.01.010 16531114

[B30] Toljamo T Kaukonen M Nieminen P Kinnula VL Early detection of COPD combined with individualized counselling for smoking cessation: a two-year prospective study Scand J Prim Health Care 28 1 41 46 10.3109/02813431003630105 PMC3440614 20331388

[B31] Dusser D Bravo ML Iacono P The effect of tiotropium on exacerbations and airflow in patients with COPD Eur Respir J 2006 27 3 547 555 10.1183/09031936.06.00062705 16507855

[B32] Jones PW Bosh TK Quality of life changes in COPD patients treated with salmeterol Am J Respir Crit Care Med 1997 155 4 1283 1289 9105068 10.1164/ajrccm.155.4.9105068

[B33] Dykstra BJ Scanlon PD Kester MM Beck KC Enright PL Lung volumes in 4,774 patients with obstructive lung disease Chest 1999 115 1 68 74 10.1378/chest.115.1.68 9925064

[B34] National Collaborating Centre for Chronic Conditions. National clinical guideline on management of chronic obstructive pulmonary disease in adults in primary and secondary care Thorax 2004 59 Suppl (1) 1 232 15041752 PMC1766028

[B35] Worster A Carpenter C Incorporation bias in studies of diagnostic tests: how to avoid being biased about bias CJEM 2008 10 2 174 175 18371254 10.1017/s1481803500009891

